# Hemorrhage from the Umbilicus

**Published:** 1893-11

**Authors:** 


HEMORRHAGE FROM THE UMBILICUS.—Lefour (*Journal de Medicine de Bordeaux*, No. 3, 1893) reports the following interesting case: The mother, a IV-para, had lost her first two children from umbilical hemorrhage, upon whose umbilical cords endarteritis could be recognized microscopically. The fourth child, four days after delivery, was observed to have a flow of bloody fluid from the ligated end of its cord, and finally a violent hemorrhage occured. Lefour applied a modification of the Emile Dubois' method, sticking five sterilized needles through the cord behind the ligature parallel in a transverse direction, beneath which he bound an elastic cord. After a few days the remains of the cord dropped off, leaving an ulcerated surface that soon healed.—*Ex.*

